# Employing Peer Outreach and Whole Health in Recovery (EMPOWER) for homeless-experienced veterans: protocol for a hybrid type 3 implementation trial

**DOI:** 10.1186/s13012-026-01499-y

**Published:** 2026-04-08

**Authors:** Daniel M. Blonigen, Justeen Hyde, Jennifer Smith, Samantha TorresTower, Tanya Podchiyska, Thomas J. Taylor, Rebecca A. Raciborski, Sharmily G. Roy, Amanda M. Midboe

**Affiliations:** 1 Center for Innovation to Implementation, VA Palo Alto Health Care System, Palo Alto, CA USA; 2 Department of Psychiatry and Behavioral Sciences, Stanford University School of Medicine, Stanford, CA USA; 3 Center for Healthcare Organization and Implementation Research, Bedford Veterans Affairs Medical Center, Bedford, MA USA; 4 General Internal Medicine, Chobanian and Avedisian School of Medicine, Boston University, Boston, MA USA; 5 Institute for Clinical and Translational Research, Baylor College of Medicine, Houston, TX USA; 6 Center for Mental Healthcare and Outcomes Research, Central Arkansas Veterans Healthcare System, North Little Rock, AR USA; 7 Department of Psychiatry, School of Medicine, University of Arkansas for Medical Sciences, Little Rock, AR USA; 8 Michael E. Debakey VA Medical Center, Houston, TX USA; 9 Department of Medicine, Baylor College of Medicine, Houston, TX USA; 10 Department of Public Health Sciences, Division of Health Policy and Management, School of Medicine, University of California–Davis, Davis, CA USA

**Keywords:** Peer support, Whole health, Homelessness, Veterans, Implementation, Facilitation

## Abstract

**Background:**

Homelessness is a national crisis in the United States, particularly in the veteran population. Due to multiple chronic conditions, homeless individuals have elevated risk for acute care service use. Engagement in primary and specialty care can mitigate this risk. Interventions grounded in evidence-based practices of peer support, patient-centered care, and whole health are effective for increasing service engagement. However, implementation of such interventions with high-acuity patients often requires multi-component strategies that are intensive and costly. This protocol paper describes a hybrid type 3 effectiveness-implementation trial of Employing Peer Outreach and Whole Health in Recovery (EMPOWER) with high-need, homeless-experienced veterans in permanent supportive housing and will evaluate the impact and cost of high-intensity (vs. low-intensity) implementation strategies on outcomes.

**Methods:**

(Aim 1) At 7 sites in the Veterans Health Administration (VA), a mixed methods pre-implementation evaluation will identify determinants and their potential impact on uptake of EMPOWER and inform modifications to the intervention and implementation strategies as needed. (Aim 2) A staircase cluster randomized design will evaluate the rollout of the implementation strategies, beginning with Audit and Feedback (low-intensity) and then switching to Implementation Facilitation (high-intensity) after 6 months. Implementation Facilitation is hypothesized to have a greater impact on the reach, effectiveness, adoption, implementation (fidelity), and maintenance of EMPOWER. (Aim 3) A budget impact analysis will estimate the average cost of implementing EMPOWER at future sites and comparative costs for implementing the low- and high-intensity strategies.

**Discussion:**

This project will provide information on the relative impacts and relative costs of strategies aimed at implementing a peer-led, patient-centered, whole health intervention for homeless-experienced veterans in permanent supportive housing. The findings will provide guidance to VA and other healthcare systems that serve the aging population of homeless-experienced veterans.

**Trial registration:**

Clinicaltrials.gov (NCT07309224).

Contributions to the literature

- High-quality care for homeless adults includes peer-based, holistic approaches. Such interventions are challenging to implement in care systems though because they require multi-pronged strategies that are costly.
- This project will evaluate the benefits and costs of using high- and low-intensity implementation strategies to increase uptake of an intervention that employs peer outreach and whole health to increase homeless-experienced veterans’ care engagement.
- Since homeless veterans in the United States utilize both Veterans Affairs and community-based care, knowledge of what works for implementing high-quality care and the associated costs are critical for public-health systems that provide a safety-net for this vulnerable population.

## Background

Homelessness is a national crisis in the United States (US). As of 2024, 23 out of every 10,000 individuals in the US is either unsheltered or unstably housed on any given night and rates of homelessness have increased in recent years [1]. Approximately 2 of 3 homeless individuals has a mental health disorder, with rates highest for substance use disorders (SUD) [2]. The relationship between homelessness and mental illness is complex and bidirectional – mental illness both increases the risk for homelessness, and mental health can be further exacerbated by the stress and sequalae of housing instability [3]. Consequently, homelessness is a robust predictor of utilization of acute care services, such as hospitalizations and emergency department visits, particularly in the veteran population [4, 5]. For these reasons, various national policies and initiatives emphasize the importance of increasing homeless individuals’ access and engagement to both primary care as well as specialty care for SUD and mental health [6].

The most effective interventions for increasing engagement in outpatient services and reducing overuse of acute care among homeless adults leverage data analytics to target and tailor services to the highest-need patients [7, 8], employ peers to help build trust and reduce stigma [9], use a holistic (vs. disease-focused) approach to incorporate patient preferences and goals [8, 10], and emphasize care coordination across an interdisciplinary team [11]. These elements form the basis of Employing Peer Outreach and Whole Health in Recovery (EMPOWER) – a multicomponent intervention to facilitate homeless-experienced veterans’ (HEVs) access and engagement in primary care and specialty care services [12, 13].

EMPOWER was developed in partnership with leaders from the Veterans Health Administration’s (VA) Homeless Program Office and is founded on three evidence-based practices (EBP): (1) peer support, (2) patient-centered care, and (3) Whole Health. Peer specialists (“peers”) are individuals with lived experience of substance use and/or mental health problems who are now in recovery and trained to support those who are actively struggling with these problems. Multiple RCTs and meta-analyses have found peer support to have a positive impact on self-reported recovery and empowerment, engagement and retention in substance use and mental health treatment, and housing stability [14, 15]. Patient-centered care is regarded by the National Academy of Medicine as foundational to effective care for high-need patients [16]. In three RCTs, peer-led, patient-centered interventions for low-income veterans with chronic health conditions increased engagement in primary care, reduced hospitalizations, and yielded a positive return on investment [17]. VA is a national leader in the implementation of Whole Health – a patient-centered care approach that focuses on empowering patients to pursue wellness in service of their life goals and values [18]. An 18-site evaluation in VA found that patients with chronic pain and mental health who received Whole Health (vs. conventional care) services had significantly greater decreases in opioid use, pain, and stress, and increases in care engagement [19].

### Barriers to implementing EBPs with vulnerable populations

An inherent challenge to implementing EBPs such as EMPOWER with vulnerable populations are the complex care needs of these patients [20]. For example, HEVs tend to have multiple, chronic physical and behavioral health conditions, along with numerous psychosocial needs (e.g., food insecurity, lack of transportation, limited digital access and literacy), which limit these patients’ capacity and motivation for self-care, goal-setting, and care engagement [21, 22]. Further, by virtue of both their level of need and eligibility for benefits, HEVs are frequently served by multiple healthcare systems and social services that are poorly integrated, leading to care fragmentation [23]. As a consequence, EBPs for high-need HEVs tend be intensive, multi-component, and require coordination across multiple stakeholders [24].

Peers may help to mitigate some of the challenges to implementing EBPs with vulnerable populations. Through their ability to build trust, role-model self-care, and provide community outreach, peers are uniquely suited to facilitating HEVs’ engagement in self-care and professional care [9]. Peers can also assist with care navigation and linkage to resources across the diverse set of services and systems available to HEVs. Successful implementation of peer-delivered EBPs, however, is dependent on several determinants such as leadership support and engagement, education and training, regular supervision, and clarity regarding the peer role [25, 26].

### Strategies to support implementation of EBPs with vulnerable populations

Education and training are generally insufficient to support the implementation of complex, multi-component interventions such as EMPOWER [27]. Implementation research on interventions similar to EMPOWER have highlighted the importance of monitoring fidelity metrics [28]. Audit and Feedback reports are an evidence-based approach to such monitoring as they include providing key stakeholders at intervention sites with summarized data about their performance relative to a standard or benchmark. This strategy is rooted in behavioral science and the belief that feedback can foster awareness of performance gaps and, in turn, motivate individuals to change their behavior to achieve desired outcomes (e.g., adoption and sustainment of an intervention) [29].

Notwithstanding the potential benefits of Audit and Feedback, more intensive strategies may be needed to further enhance implementation of complex EBPs that serve high-acuity populations [27]. One example, Implementation Facilitation [30], is a collaborative strategy in which trained individuals work with organizations or teams to support the adoption, implementation, and sustainment of an EBP. It is a dynamic process that involves tailored guidance, problem-solving, technical assistance, and capacity-building activities to address specific barriers and leverage facilitators of change [31]. To this end, external facilitators often collaborate with local champions and/or internal facilitators to bring expertise regarding the implementation processes and have transferable knowledge in relevant clinical and behavior change models that inform implementation of the EBP. For example, external facilitation in the form of pre- and post-implementation site visits, regular calls, and interactive support of performance data was shown to be effective for the implementation of peer services in multidisciplinary teams in VA [32].

### Objectives and aims

In this paper, we describe the protocol for a multi-site trial to evaluate the implementation of EMPOWER with high-need, HEVs in the Housing and Urban Development (HUD) and VA Supportive Housing (VASH) program. HUD-VASH is the largest permanent supportive housing program in the US, housing over 180,000 veterans since fiscal year 2012. HUD-VASH has two goals: (a) assist HEVs with obtaining permanent supportive housing, and (b) facilitate access to healthcare and psychosocial services for veterans to maintain housing [33]. The overarching objectives of the current trial are to implement and evaluate EMPOWER at 7 VA sites by (Aim 1) identifying pre-implementation barriers and facilitators to make fidelity-consistent modifications to the intervention and to refine implementation strategies for the local settings; (Aim 2) using a hybrid type 3 staircase cluster randomized trial to test the implementation of EMPOWER through tailored low-intensity (Audit and Feedback) and high-intensity strategies (Implementation Facilitation); and (Aim 3) conducting a multi-payer economic analysis. Implementation outcomes will be evaluated using the Reach, Effectiveness, Adoption, Implementation, and Maintenance (RE-AIM) framework [34]. We hypothesize that the high-intensity implementation strategy of Implementation Facilitation will have a greater impact on all RE-AIM outcomes than the lower-intensity strategy of Audit and Feedback.

**Fig. 1:**
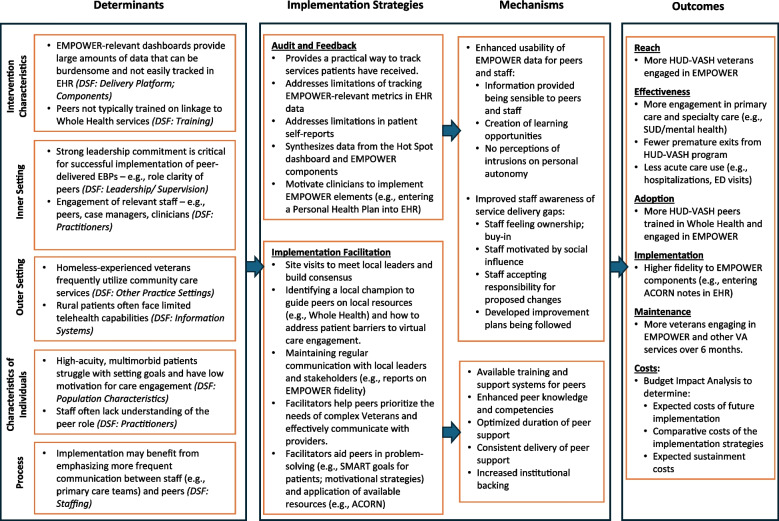
Implementation research logic model for EMPOWER in HUD-VASH programs

Figure 1 provides the implementation logic research model [35], which guided the planning of the current trial. Examples of key determinants are provided, which informed the selection of the implementation strategies to be tested. The determinants were identified from the aforementioned literature, with relevant domains from the Consolidated Framework for Implementation Research (CFIR) [36] and the Dynamic Sustainability Framework (DSF) [37] determined from a scoping review of implementation studies in vulnerable populations [38]. For example, leadership engagement (CFIR: Inner Setting) is a critical determinant of successful implementation of peer-delivered EBPs as well as health-focused interventions for vulnerable populations [26], suggesting that meeting with local opinion leaders will be a key activity in the design of the Implementation Facilitation strategy in the current trial. The model also specifies how the RE-AIM outcomes will be measured and the hypothesized mechanisms of how the implementation strategies will impact those outcomes.

## Methods

### Aim 1

To refine the implementation approach of EMPOWER at project sites, a convergent parallel mixed methods pre-implementation evaluation will be conducted to identify a local understanding of key determinants and their potential impact on uptake of the intervention [39]. The design of this evaluation is guided by CFIR and DSF [40]. An integration of these two frameworks was previously applied in a mixed methods, multi-site trial to identify determinants to implementation of an EBP for HEVs in the HUD-VASH program [40]. At each site, we will conduct qualitative interviews with 5–10 key informants – e.g., HUD-VASH case managers, peers, and supervisors. Interviews will be conducted via Microsoft Teams, audio-recorded with permission, and follow a semi-structured interview guide. De-identified audio-files of all interviews will be transcribed and analyzed using a rapid qualitative approach, which has been found to yield equivalent findings to more in-depth, time-intensive methods [41]. These analyses will be guided by the Planning for and Assessing Rigor in Rapid Qualitative Analysis (PARRQA) framework, which provides criteria to ensure rigorous planning, conduct and evaluation of qualitative data (e.g., summary template development, matrix analysis) [42].

To complement the qualitative data, the key informants will also be asked to complete three measures of acceptability, appropriateness, and feasibility of the proposed intervention. Each measure consists of four items. The Acceptability of Implementation Measure will measure the extent to which the key informants perceive EMPOWER to be agreeable or satisfactory [43]. The Intervention Appropriateness Measure will be used to assess the perceived fit, relevance, and compatibility of EMPOWER with the practice settings [43]. The Feasibility of Intervention Measure will measure the extent to which EMPOWER can be successfully implemented within the settings [43].

### Aim 2

#### Design

**Fig. 2:**
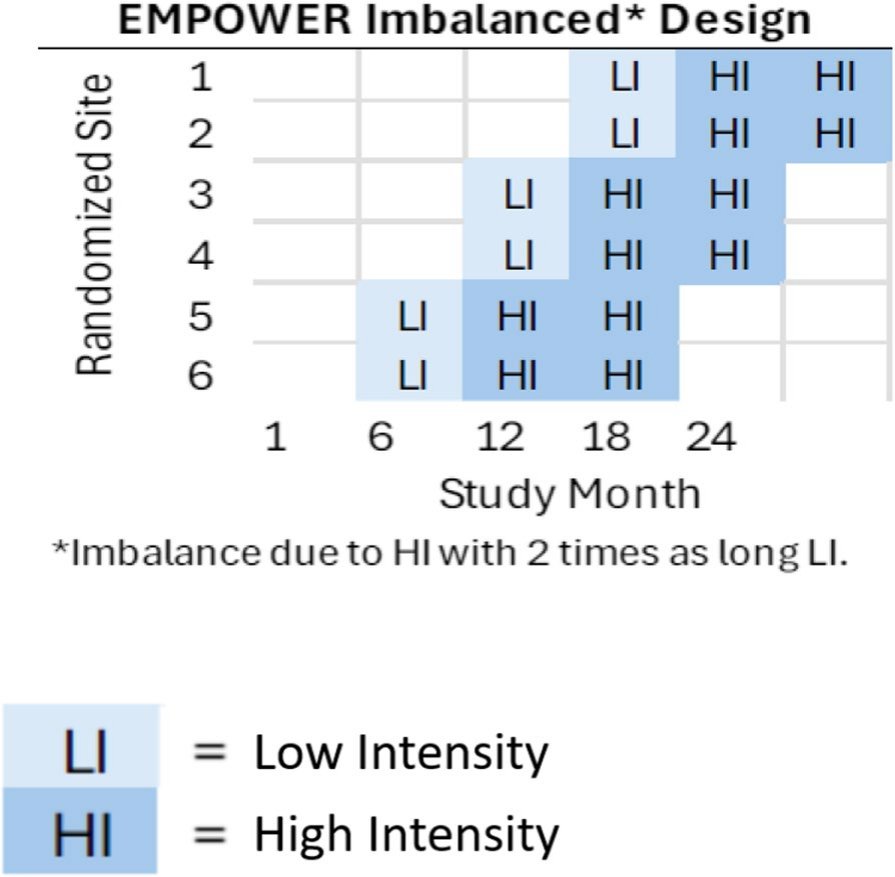
Staircase cluster randomized trial analytic approach

We will conduct an adaptation of the multi-site hybrid type 3 cluster randomized staircase stepped-wedge trial, evaluating the implementation of EMPOWER while gathering data on its effectiveness. The staircase design is an adaptation to stepped wedge designs (SWD) that accommodate more flexible implementation (i.e., less burden to sites and clinicians). At the same time, it offers robust statistical efficiency given that statistical information is often strongest around time periods in the SWD when switching from one implementation strategy to another [44]. For EMPOWER, of interest is the effect of switching from Low Intensity (LI) to High Intensity (HI) implementation strategies. An imbalanced design [45] will be employed in which sites will spend twice as long in HI than LI (see Fig. 2). The use of an imbalanced design further accommodates the anticipated needs of sites to be enrolled in EMPOWER.

#### Sample

At each implementation site, eligible patients will be identified from VA’s Homeless Registry “Hot Spot” reports, which use real-time data on acute care service utilization to identify high-need, housing-insecure patients [7, 46]. These reports identify veterans on the VA Homeless Registry (i.e., those who had received VA housing services in the past two years) who had ≥ 1 hospital admissions and/or ≥ 2 ED visits in the past quarter of the fiscal year [47]. Reports are housed on VA’s Support Services Center (VSSC) website with data primarily drawn from the Corporate Data Warehouse (CDW). Data for the reports are updated nightly and separate reports are generated for those who are (and are not) enrolled in VA primary care. From these reports, we will identify patients at each implementation site who are (a) currently enrolled in HUD-VASH, (b) have a mental health and/or SUD diagnosis, and (c) have not been engaged in either primary care or mental health during the quarter that they were flagged on the report. Approximately 585 patients across the target sites are estimated to be eligible to receive EMPOWER during the project period.

Power was assessed by testing a total of 448,560 plausible scenarios for a binomial outcome (e.g., patient reach) with the effect size (θ) of central interest being the mean difference in proportion of patients reached when moving from LI to HI. We assessed the necessary number of patients to achieve Power = 0.80 at α = 0.05, by including a random intercept for each cluster in the analyses (τ) is ∈ {0.01, 0.05, 0.10, 0.15} with θ ranging from 0.01 to 0.20 increase in number of patients reached and an autoregressive correlation of 0.7 between LI and HI implementation periods. Under a variety of plausible scenarios, we assessed power in relation to sample sizes ranging from a total N of 200–1000 patients (the plausible lower and upper limits, respectively, of patients eligible for EMPOWER). We observed Power = 0.80 to be achieved in sample size ranges between 227 and 278 patients.

#### Intervention

**Fig. 3:**
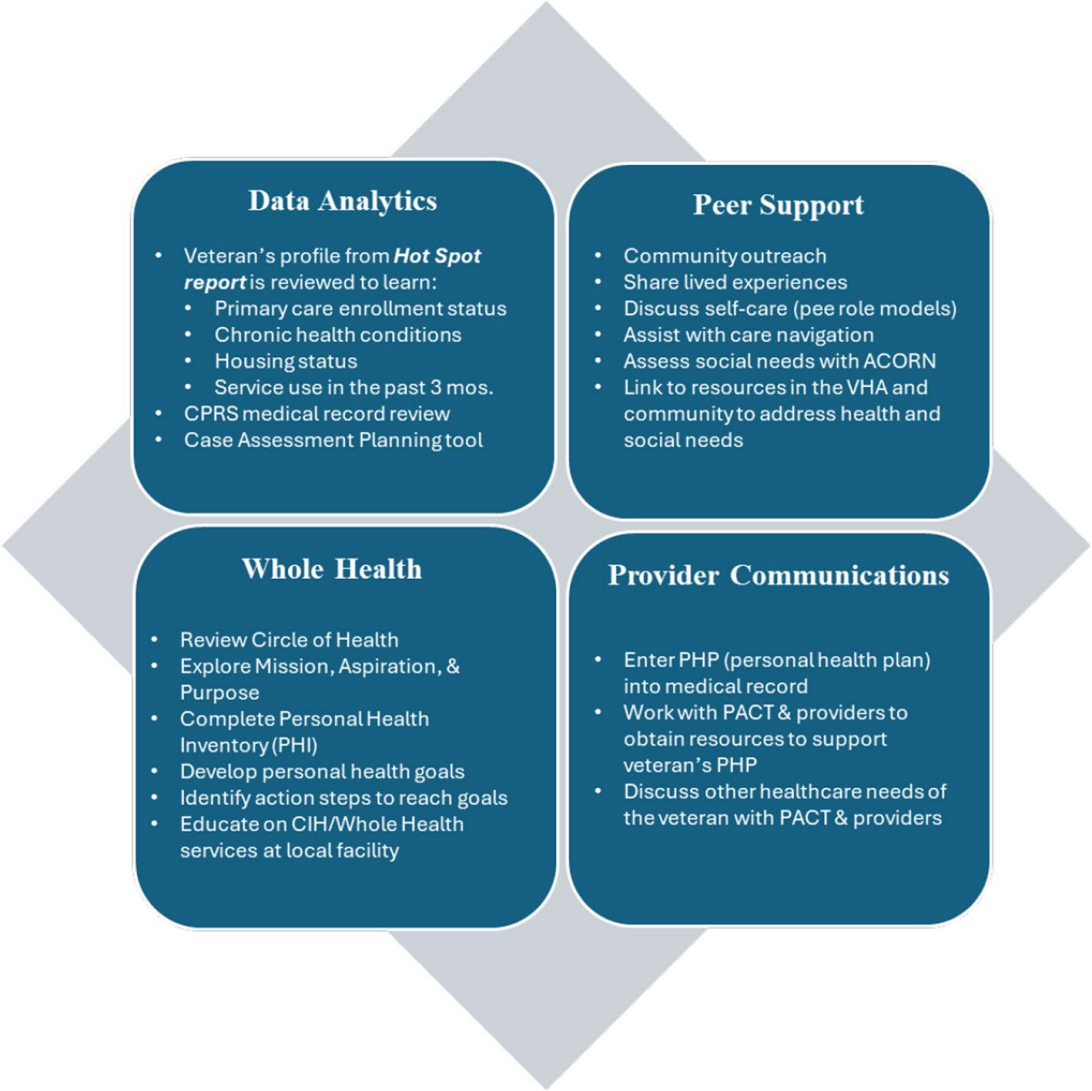
Core components of EMPOWER

Figure 3 outlines the four core components of EMPOWER: data analytics, peer support, use of and connection to Whole Health resources, and communication with veterans’ healthcare teams [12, 13]. Data analytics: HUD-VASH case managers identify high-need, HUD-VASH veterans on the Homeless Registry Hot Spot Report. Veterans’ profiles are reviewed to learn about their chronic health conditions, housing status, acute care use, and engagement in supportive care – e.g., primary care, outpatient SUD. Peer support: HUD-VASH peers meet with identified veterans for up to six months, averaging once-per week sessions for the first three months (via phone, video, or in-person), with step-down in frequency as veterans begin to engage in services and reach their goals. By sharing lived experiences, role modeling, and assisting with care navigation, peers build trust, reduce stigma, and motivate patients to engage in services for their health and social needs. This process is structured through a social needs screener – Assessing Circumstances & Offering Resources for Needs (ACORN) [48]. Whole Health: During sessions, peers use a Whole Health approach to collaboratively develop personal health goals that align with the veteran’s priorities and values. Peers help veterans with completing a Personal Health Inventory and developing a Personal Health Plan tailored to their priorities for self- and professional care. Peers help veterans identify action steps to implement their plan, review progress, and problem-solve barriers. Peers also educate the veteran on locally available Whole Health and Complementary and Integrative Health services and provide a warm handoff to a Whole Health Coach, as requested. Provider communications: Peers communicate with a veteran’s care providers to share the veteran’s personal health goals. To this end, peers enter the Personal Health Plan note into the electronic health records (EHR) and include the veteran’s primary care team (if they are enrolled in primary care) and any other providers the veteran identifies as important to their professional care. Peers may communicate with providers to discuss the Personal Health Plan and learn if there are care needs of the veteran for which the peer may be able to help.

#### Implementation strategies

All sites will receive education and training in the core components of EMPOWER. During the first three months of the project, peers at each site will attend a VA-sponsored, 3-day virtual training in Whole Health. Peers will also be invited to join monthly Whole Health Community of Practice calls hosted by the VA office that supports the implementation of Whole Health VA-wide (Office of Patient Centered Care & Cultural Transformation) and be given access to a SharePoint site housing Whole Health educational materials. Following the Whole Health trainings, EMPOWER virtual trainings will be offered by the first author (DMB). These trainings will cover EMPOWER Manual content, including using Hot Spot reports and completing EHR note templates and Fidelity Monitoring forms.

For the LI strategy of Audit and Feedback, consistent with recommended guidance, data will be provided to participating sites regarding their performance, relative to pre-defined goals, and delivered in multi-modal formats (e.g., visual graphics, written reports) in repeated cycles [49]. Specifically, HUD-VASH peers and supervisors at each site will be emailed monthly automated reports on EMPOWER fidelity data from the EHR (e.g., % of HUD-VASH patients with a Personal Health Plan note) as well as aggregated data on treatment engagement via the Hot Spot dashboard (e.g., % of HUD-VASH veterans with an SUD diagnosis who received SUD specialty care in the past month). The monthly reports will include tailored action item recommendations based on the local site’s performance. Under this LI strategy, sites will not be provided with interactive support to review these reports.

For the HI strategy of Implementation Facilitation, this approach will be tailored to the unique context, goals, and challenges of each site. Consistent with previous implementation work with peers in VA [32], we will employ Implementation Facilitation activities, as described in Table 1. As noted in the implementation research logic model in Fig. 1, the selection of these activities for the current trial was guided by the determinants identified from the literature in terms of implementation of interventions similar to EMPOWER as well as other health-focused interventions in vulnerable populations [38].

**Table 1:** Implementation facilitation activities for EMPOWER

Activity	Description
Engagement with the facilitation team	Monthly calls between the external facilitators and site-specific internal facilitators and local champions (if one is identified during pre-implementation)
Audit and feedback	Reports on EMPOWER fidelity data provided monthly to sites’ internal facilitators
Meeting with local opinion leaders	During pre-implementation site visits, meeting with local leaders who are familiar with the site’s organizational procedures and culture, to build consensus and tailor the strategy to the local setting
Problem-solving	Bi-weekly video calls and ad hoc meetings between the Facilitator and peers at a site to troubleshoot challenges such as identifying SMART (Specific, Measurable, Actionable, Realistic, Time-bound) goals with veteran patients
Technical assistance	Assisting HUD-VASH case managers on the use of the Hot Spot reports, and peers on the use of the Personal Health Inventory and ACORN

#### Outcome measurement

The RE-AIM framework [34] will be used evaluate the implementation of EMPOWER. Reach will be measured in terms of the number of patients who are willing to receive EMPOWER, out of all patients that are estimated to be eligible at potential sites. Effectiveness will be operationalized as number of outpatient visits in primary and specialty visits for mental health/SUD care, engagement in Whole Health services offered by sites [50], and premature exits from the HUD-VASH program. Additional patient-level outcomes will include rates of suicide screening and hospitalizations and emergency department visits, particularly for mental health or substance use. All service use variables will be measured in terms of receipt in VA and in the community using data from CDW. Adoption will be measured in terms of the number of peers in HUD-VASH at sites that are trained in EMPOWER and initiate the intervention with eligible patients at their site. Implementation will be operationalized in terms of fidelity to the various elements of EMPOWER at the site level, as measured via activity logs embedded in the EHR and electronic data capture logs (e.g., Qualtrics) to document type and length of encounters. Maintenance will be measured by the number of patients who are continuing to engage in EMPOWER and other VA services over the duration of the intervention (i.e., 6 months). To supplement the quantitative data used to operationalize the RE-AIM domains, we will conduct semi-structured qualitative interviews by phone with six key informants at each site – e.g., HUD-VASH peers, supervisors, and case managers (n = 36). The interview questions and format will be aligned with the qualitative data collection methods described above in Aim 1.

#### Analysis

We hypothesize the HI (vs. LI) implementation strategy will have a greater impact on the RE-AIM outcomes*.*For each outcome, we will specify Generalized Linear Mixed Effects models (GLMMs) using unstructured correlation matrices for random effects to flexibly account for variation over time within patients and variation over time within cluster sites with patients clustered within sites [51]. Individual patients may be observed in both LI and HI (thus unbalanced within clusters over time, with some observed multiple times and others at only one stage of implementation). Random effects components of the GLMM will account for this unbalanced panel nature of patient eligibility and site volume in any given time point [51]. In addition to the staircase design’s randomization providing some protection from confounding influences, we will include both patient- and site-level covariates to improve efficiency of the treatment estimates. At the patient level, we will account for demographics to explore potential variations in effectiveness based on these factors (interaction terms). At the site level, we will account for covariates that may represent confounders despite randomization, including site patient volume normalized by providers at the site (site capacity and time to meet patient needs) and staffing changes. Communication with the sites will enable us to identify other factors affecting implementation that we may want to account for in models that may affect the relative success of HI over LI, or vice versa.

Because Reach can be assessed as counts or rates of patients, we will specify Poisson GLMMs. For predicting individual reach to a veteran (a binary outcome), Poisson GLMMs with robust standard errors will be fit to allow convergence and provide accurate treatment estimates [52]. Because EMPOWER will afford more opportunity time in HI than LI to achieve outcomes, the Poisson GLMM approach allows a facile offset term to account for the different opportunity lengths for LI and HI conditions. At the patient level, time dynamics can be nuanced because care-seeking is dynamic and may represent residual confounding for which site-level randomization cannot account. Specifically, patients who seek more care may have a greater dosage of exposure to the condition the site is in at a particular time. Accordingly, we will also account for the longitudinal number of encounters (via stop codes) a patient has in the site and offset those encounter opportunities in the Poisson GLMM. This effort is aimed at improving precision of estimates of LI and HI effects in the models.

### Aim 3

To examine the implementation costs of EMPOWER, we will use a sequential mixed-methods design where an initial qualitative phase informs customization of the typical quantitative approach of a budget impact analysis [53]. Consistent with Eisman and colleagues’ work [54], we will conduct semi-structured interviews to identify stakeholder perspectives, which will guide the level of detail to be employed in micro-costing – e.g., including different payers’ relative priorities on how they may use cost information for implementation planning. We will use rapid qualitative methods, as described above in the Methods section for Aim 1. Interview participants will be drawn from a Learning Community of regional and national VA stakeholders associated with project and other medical center leadership.

Quantitative data collection and a budget impact analysis (BIA) will then proceed, informed by the needs of operational partners and clinical leadership. Budget impact analyses are conducted from the perspective of the entity that pays for the intervention (“payer”). Because implementation of EMPOWER requires resource allocation across different contexts, we will adopt three payer perspectives, all of which will be represented in the qualitative phase: (1) HUD-VASH – the relevant national program office, (2) Veteran Integrated Service Networks, and (3) VA facilities. Consistent with best-practices for conducting a BIA [53], we will include costs necessary to deliver EMPOWER (e.g., peer time to conduct veteran meetings), as well as ensure it is appropriately delivered in practice (e.g., Whole Health trainings). Costs incurred only for the evaluation of the implementation (e.g., time collecting payer perspectives) and fixed costs that later sites would not incur (e.g., development of reporting tools) will be excluded.

Costs of specific elements of EMPOWER (e.g., staff time) that have existing values will be obtained using the VA’s Managerial Cost Accounting Office’s data in CDW [55]. Because implementation strategies do not have a standard value, we will estimate their cost using activity-based costing from time use data gathered using an electronic data capture system [56]. Activity-based costing emphasizes the role of staff effort devoted to different activities within a strategy. Where indicated by the Learning Community, additional elements of strategy costs may be micro-costed as well. The BIA will report (1) expected implementation cost for implementation of EMPOWER at future sites, (2) comparative implementation cost for the LI and HI strategies, and (3) expected sustainment cost over a relevant budget period. Indexing time, from the beginning of implementation of EMPOWER, all costs will be assigned to a stage of implementation (pre-, during-, post-) using standard methodology [57]. Based on Learning Community feedback, we will consider additional perspectives and cost analysis methods to our approach (e.g., implementation cost-effectiveness analysis, analysis of variation in costs by effort level of involved personnel).

## Discussion

The hybrid type 3 trial described in this protocol paper will contribute to the broader literature on the promise and pitfalls of implementing interventions for vulnerable populations in healthcare settings in the US. Due to their multimorbidity and extensive social needs, HEVs are a particularly vulnerable patient population such that high-quality care often necessitates intensive, multicomponent interventions that can be costly to implement and sustain. Notably, the cost of care for HEVs is likely to increase in future years due to the aging nature of this population. That is, rates of homelessness in the US are expected to triple among those 65 years and older in the next decade [58]. Consistent with this, a recent analysis of a national cohort of high-need HEVs from the VA’s Homeless Hot Spot Registry found that the proportion of patients age ≥ 65 almost doubled from 2016 to 2022 (14% vs. 27%) [22]. An added complication to care efforts for this population is that many of these patients utilize community-based services, particularly acute care for psychiatric reasons [59], thus necessitating integration and coordination of care with VA services. For these reasons, knowledge about the relative impacts and relative costs of strategies aimed at implementing interventions such as EMPOWER for HEVs will be valuable for the larger healthcare system in the US.

The current trial will also provide valuable insights into the implementation of both peer-led and Whole Health-focused interventions more generally. Nationally, the peer workforce in the US has expanded considerably in recent years. For example, 77% of new staff positions in Certified Community Behavioral Health Clinics in 2024 were peers [60], and the majority of US states now reimburse for peer services through Medicaid [61]. However, high rates of burnout and turnover are well-documented challenges to implementation of this workforce [62], which often stems from inadequate training and supervision [62, 63]. For these reasons, Implementation Facilitation may be an ideal strategy to support implementation of new peer-led interventions, such as EMPOWER. The findings from the current trial will add to the small body of research on implementation of this workforce in large healthcare systems [32]. In terms of Whole Health care, health and wellness coaching, personal health planning, and various complementary and integrative therapies (e.g., acupuncture, meditation, yoga) have increased in the past decade [64]. As the US healthcare system continues to shift from a disease-centered model of care to one focused on supporting patients’ overall health and wellness [18], additional research is needed to understand what enables and hinders this model of care. The current trial in particular will provide novel insights into the impacts and costs associated with integrating peer support and Whole Health care.

One limitation of the current trial is the generalizability of findings to the implementation of EMPOWER and similar interventions outside the VA healthcare system. Though peers and other homeless program staff tasked with implementing EMPOWER may be familiar with the community-based services available to HEVs and will support linkage of these patients to those services as appropriate, the current trial is ultimately centered on the VA care system. In addition, though data on utilization of VA-paid services will be available for HEVs who receive EMPOWER in the current trial, community-based services that are not paid for by the VA will not be captured; thus, measurement of outpatient and inpatient care for HEVs may be an underestimate.

## Conclusion and impact

The current project will provide evidence of the impact and costs associated with using low- and high-intensity strategies to implement EMPOWER – a multi-component intervention for HEVs grounded in the evidence-based practices of peer support, patient-centered care, and Whole Health. By focusing on increasing care engagement in HEVs, EMPOWER can offer a high-quality care model for a large population of HEVs in the US. Accordingly, the findings will provide guidance to VA and other healthcare systems that frequently serve this high-need and vulnerable patient population.

## Data Availability

The datasets generated and/or analyzed during the current project will not be publicly available but will be available from the corresponding author on reasonable request.

## Abbreviations

ACORN: Assessing Circumstances & Offering Resources for Needs
BIA: Budget Impact Analysis
CDW: Corporate Data Warehouse
CFIR: Consolidated Framework for Implementation Research
DSR: Dynamic Sustainability Framework
EHR: Electronic Health Record
EBP: Evidence-based practices
EMPOWER: Employing Peer Outreach and Whole Health in Recovery
GLMM: Generalized Linear Mixed Effects Models
HEV: Homelessness-experienced veterans
HI: High Intensity
HUD: Housing and Urban Development
LI: Low Intensity
PARRQA: Planning for and Assessing Rigor in Rapid Qualitative Analysis
RCT: Randomized Control Trial
RE-AIM: Reach, Effectiveness, Adoption, Implementation, and Maintenance
SUD: Substance Use Disorder
SWD: Stepped Wedge Designs
VA: Veterans Health Administration
VASH: VA Supportive Housing
VSSC: VA Support Services Center
